# *Toxoplasma gondii* HOOK-FTS-HIP Complex is Critical for Secretory Organelle Discharge during Motility, Invasion, and Egress

**DOI:** 10.1128/mbio.00458-23

**Published:** 2023-04-24

**Authors:** David J. Dubois, Sylia Chehade, Jean-Baptiste Marq, Kannan Venugopal, Bohumil Maco, Albert Tell I. Puig, Dominique Soldati-Favre, Sabrina Marion

**Affiliations:** a Department of Microbiology and Molecular Medicine, University of Geneva, Geneva, Switzerland; b University Lille, CNRS, Inserm, CHU Lille, Institut Pasteur de Lille, U1019—UMR 9017—CIIL—Center for Infection and Immunity of Lille, Lille, France; c Wellcome Centre for Integrative Parasitology, Institute of Infection, Immunity, and Inflammation, University of Glasgow, Glasgow, United Kingdom; University of Pittsburgh

**Keywords:** Apicomplexa, *Toxoplasma gondii*, traffic, micronemes, invasion, egress, motility, exocytosis, endosomal transport, HOOK, Fused Toes, FTS

## Abstract

Members of the Apicomplexa phylum possess specialized secretory organelles that discharge, apically and in a timely regulated manner, key factors implicated in parasite motility, host cell invasion, egress and subversion of host cellular functions. The mechanisms regulating trafficking and apical docking of these secretory organelles are only partially elucidated. Here, we characterized two conserved endosomal trafficking regulators known to promote vesicle transport and/or fusion, HOOK and Fused Toes (FTS), in the context of organelle discharge in Toxoplasma gondii. TgHOOK and TgFTS form a complex with a coccidian-specific partner, named HOOK interacting partner (HIP). TgHOOK displays an apically enriched vesicular pattern and concentrates at the parasite apical tip where it colocalizes with TgFTS and TgHIP. Functional investigations revealed that TgHOOK is dispensable but fitness conferring. The protein regulates the apical positioning and secretion of micronemes and contributes to egress, motility, host cell attachment, and invasion. Conditional depletion of TgFTS or TgHIP impacted on the same processes but led to more severe phenotypes. This study provides evidence of endosomal trafficking regulators involved in the apical exocytosis of micronemes and possibly as a consequence or directly on the discharge of the rhoptries.

## INTRODUCTION

Toxoplasma gondii is one of the most ubiquitous zoonotic parasites, infecting a wide range of animals and about a third of the human population. T. gondii infection is usually asymptomatic but can be responsible for neonatal malformations or abortion during primary infection of pregnant women (1). Moreover, this parasite persists chronically and is a threatening opportunistic pathogen in immuno-incompetent individuals.

T. gondii belongs to the phylum of Apicomplexa characterized by a set of unique apical secretory organelles called the micronemes and rhoptries that sequentially discharge their contents and enable parasite invasion and dissemination. Notably, micronemes release adhesin complexes that critically participate in parasite motility and invasion, as well as perforins to egress from infected cells (2). Host cell invasion relies on the formation of a transient adhesive structure between the parasite and the host plasma membrane called the moving junction (MJ) (3). The MJ is formed via the interaction between the microneme protein, AMA1, secreted at the parasite apical surface, and the rhoptry-neck protein complex RON2/4/5 inserted into the host plasma membrane (4). Several rhoptry proteins (ROPs) contained in the bulb of the organelle contribute to the formation of the parasitophorous vacuole (PV) in which the parasite multiplies and to the subversion of host functions to ensure parasite survival (5).

While the signaling cascades leading to microneme exocytosis have been well characterized (2), the mechanisms regulating organelle trafficking and apical positioning prior to exocytosis are not elucidated. T. gondii possesses a noncentrosomal microtubule organizing center positioned at the apex of the parasite that consists of a ring-shaped structure, named the apical polar ring (APR), from which emerge the subpellicular microtubules. Anchored to the APR, the conoid is composed of a packed spiral of tubulin fibers, a pair of intraconoidal microtubules at its center and two preconoidal rings (PCRs) at the top (6–8). The conoid is a dynamic organelle which is retracted in replicating intracellular parasites but extrudes upon rise in intracellular calcium (Ca^2+^) levels in activated parasites (9, 10). The micronemes appear to be stored along the subpellicular microtubules near the APR and transported through the cone of the conoid prior to fusion with the plasma membrane at the apical tip (11, 12).

To date, few proteins involved in microneme apical positioning or their trafficking along the subpellicular microtubules have been identified. Kinesin A and Apical Polar Ring 1 (APR1) promote APR stability and subpellicular microtubule organization thereby contributing to microneme exocytosis (11). Ring 2 (RNG2), a component of the APR, was also shown to function in the constitutive and cGMP-stimulated secretion of micronemes (12). Depletion of the dynein light-chain 8a (DLC8a), which localized at the apical tip of the parasite (13), leads to reduced microneme exocytosis and a partial defect in the apical positioning of rhoptries. Overall, depletion of DLC8a strongly impacts on invasion and microneme secretion but only moderately affects motility, egress, and host cell attachment (14). Finally, centrin 2, which localizes to the PCRs but not only, is critical to microneme exocytosis thereby contributing to parasite motility, invasion, and egress (14, 15).

In higher eukaryotes, organelle trafficking and positioning rely on their interaction with the cytoskeleton through trafficking regulators, notably Rab GTPases. For instance, Rab11 interacts with the molecular motor myosin V to regulate recycling endosome transport on actin filaments (16) and with the kinesin KIF13A to regulate endosome tubule formation and transport on microtubule tracks (17). Rab-mediated cargo anchoring to cytoskeleton structure can be either direct or indirect, in the latter case requiring adaptor molecules such as the HOOK protein family. The HOOK protein family consists of broadly conserved proteins that contribute to endosomal trafficking. In Ustilago maydis and Aspergillus nidulans, HOOK participates in early endosome transport by interacting with dynein and kinesin-3 motors (18–20). Most eukaryotes encode a single HOOK isoform; however, mammals have three paralogues, which appear to have specific functions. HOOK1 is implicated in spermiogenesis and endosomal trafficking in neurons (21, 22). HOOK2 protein associates with the centrosome and contributes to the establishment and maintenance of centrosome structure, function, and homeostasis (23–25). HOOK3 was originally described as a Golgi body-associated protein that contributes to Golgi integrity (26) and more recently to kinesin-mediated vesicular transport (27). Mammalian HOOKs have been implicated in a variety of endosomal trafficking pathways (28–33). HOOK1 interaction with Rab7 and Rab9 suggested a regulatory function in late endosomal compartment via an interaction with the homotypic vacuolar protein sorting complex (29, 31). In a distinct study, mammalian HOOK1 was shown to be also implicated in the recycling of specific clathrin-independent endocytic cargos via endosomes decorated with Rab11 and Rab22 (30). Moreover, HOOK functions as a multiprotein complex together with Fused Toes (FTS), an inactive variant of an E2 ubiquitin-conjugating enzyme, and FTS/hook interacting protein (FHIP). This complex, called the FHF complex, coordinates vesicle tethering and transport onto microtubule tracks (19, 29, 31, 33). Recently, a study performed in hippocampal neurons suggested that HOOK1 and HOOK3 are involved in Rab5-mediated endosome retrograde motility in axons by binding to cargo through C-terminal interactions with FTS and FHIP proteins (34), similar to what has been described in fungi (19). The FHF complex also interacts with the clathrin subunit AP-4 and promote endosome clustering at the perinuclear region (33).

Here, we characterized HOOK in T. gondii and found that it interacts with TgFTS and with a coccidian-specific HOOK interacting protein (TgHIP). Localization and functional dissection of the components of T. gondii HOOK-FTS-HIP complex revealed that they critically and differentially contribute to microneme positioning and exocytosis as well as rhoptry discharge.

## RESULTS

### TgHOOK is localized at the apical tip of *Toxoplasma gondii*.

Proximity-dependent biotin identification (BioID) was applied to AMA1 with the biotin ligase fused to the cytoplasmic tail of this transmembrane microneme protein. The proteins identified are potentially localized to the cytosolic surface of the micronemes (35–37). Among the hits, TGGT1_289100 stood out as closely related to HOOK, a conserved eukaryotic endosomal trafficking protein (see Table S1 in the supplemental material). In parallel, TGGT1_289100, named T. gondii HOOK (TgHOOK), was identified as a preferential partner of Rab11A, based on affinity pulldown assays performed with a GTP-bound active form of Rab11A (see Table S1). Rab11A was previously reported to be implicated in parasite cytokinesis (38). We demonstrated that Rab11A also promotes the secretion of dense granules in replicating parasites, as well as microneme secretion in extracellular parasites correlating with a drastic defect in parasite adhesion, motility, and host cell invasion (39). Interestingly, pulldown assays performed with purified active GTP-bound Rab5A, Rab7, Rab11A, and Rab11B revealed that TgHOOK preferentially interacts with Rab11A and to a lesser extent with Rab11B, but not with Rab5 and Rab7, suggesting a putative regulation of Rab11A-dependent trafficking pathways by TgHOOK (see Fig. S1A).

10.1128/mbio.00458-23.1 FIG S1 (A) Western blot showing the results of the GST Rab-pulldown performed using HOOK-HA parasite lysates. TgHOOK was preferentially pulled down with GST-Rab11A preloaded with GDP or GTPγs and to a lesser extend to GST-Rab11B/GTPγs but not with GST-Rab5A/GTPγs and GST-Rab7/GTPγs. IN, input; E, elution (from beads). The lower panel indicates GST and GST-Rab protein levels revealed by Coomassie blue staining. (B) Cell cycle transcriptional expression profile of TgHOOK compared to MIC2, MIC3, and APH providing representative examples of microneme proteins. (C) IFA showing that TgHOOK partially colocalizes with Rab11A positive vesicles in intracellular replicating parasites. Scale bar, 2 μm. Download FIG S1, TIF file, 1.3 MB. Copyright © 2023 Dubois et al. 2023 Dubois et al. https://creativecommons.org/licenses/by/4.0/ This content is distributed under the terms of the Creative Commons Attribution 4.0 International license.

10.1128/mbio.00458-23.8 TABLE S1 Table showing the results of AMA1-tail BioID fusion immunoprecipitation, followed by MS analysis with indication of the total number of identified peptides. The AMA1-tail BioID fusion strain consists of the integration of the biotin ligase directly in frame with the C terminus of the endogenous locus. This construct was expressed in RHΔKu80. Table showing the MS results for the GST pulldown experiment using a GDP-bound inactive or GTP-bound active Rab11A proteins. TgHOOK preferentially interacts with the GTPγS-bound active form of Rab11A. Download Table S1, XLSX file, 0.02 MB. Copyright © 2023 Dubois et al. 2023 Dubois et al. https://creativecommons.org/licenses/by/4.0/ This content is distributed under the terms of the Creative Commons Attribution 4.0 International license.

**FIG 1:**
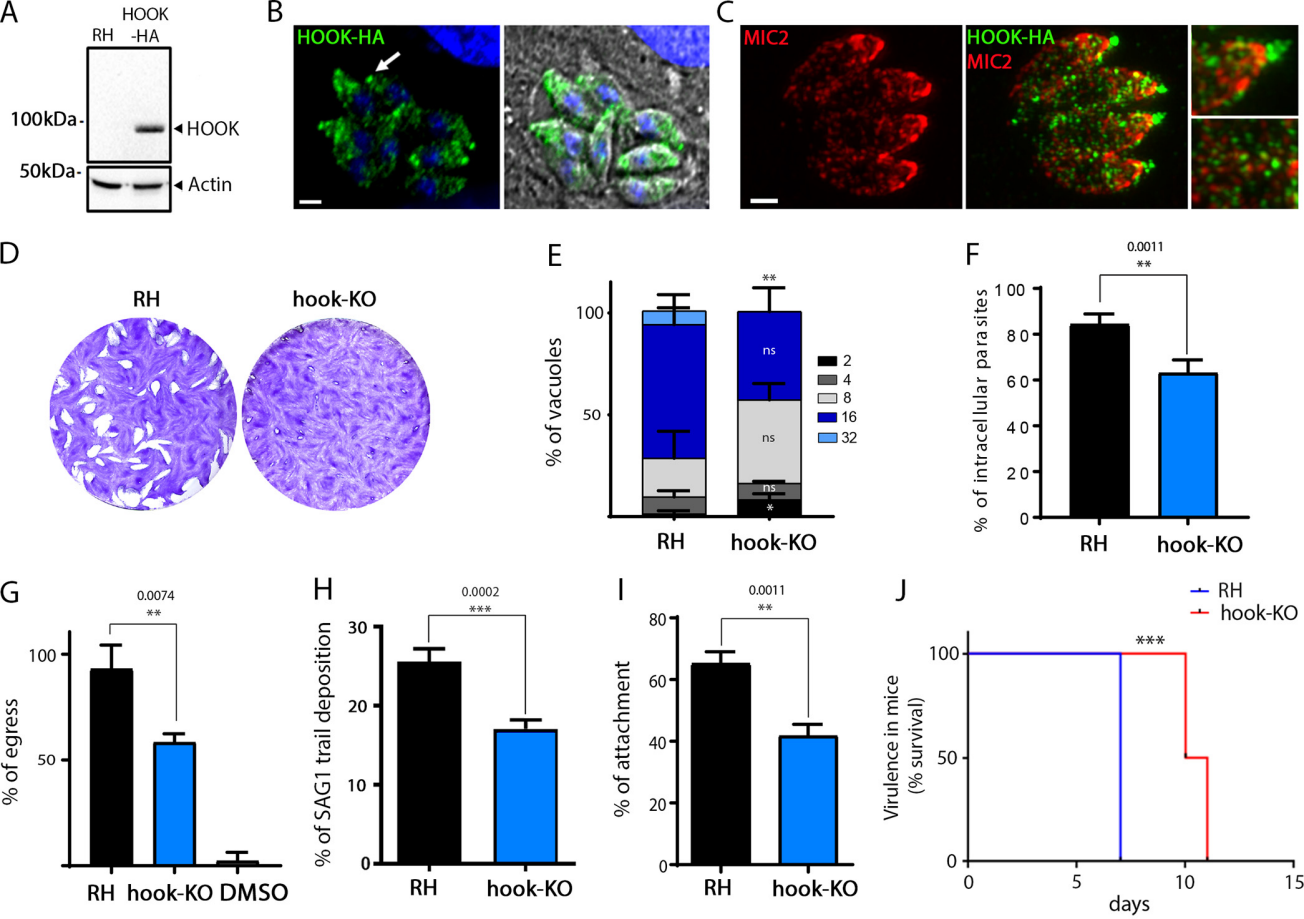
TgHOOK partially contributes to host cell attachment, invasion, and egress from infected cells. (A) Western blot (WB) revealing the expression of HA-tagged TgHOOK at the predicted size of 79 kDa, which was not detected in the RH control strain. Act1, actin (loading control). (B) IFA performed in intracellular replicating parasites shows TgHOOK localization at the apical tip (arrow) and in numerous vesicles enriched at the apical region of the parasite. (C) Costaining with the MIC2 protein shows that the TgHOOK does not coincide with micronemes. Scale bars, 2 μm. (D) Hook-KO parasites display a significant impairment in the lytic cycle, as shown by the smaller lysis plaques compared to the parental line (RH). (E) Quantification of the percentage of vacuoles containing 2, 4, 8, 16, or 32 parasites at 30 h postinfection for the parental (RH) and hook-KO lines. Student *t* test: **, *P* < 0,01; ***, *P* < 0,001. (F) Percentage of parasites (RH and hook-KO lines) that have invaded host cells (**, *P* = 0.0011). (G) Percentage of parasites (RH and hook-KO lines) that have egressed from host cells after BIPPO stimulation (**, *P* = 0.0074). DMSO treatment was used as a negative control. (H) Percentage of RH and hook-KO parasites displaying a SAG1-positive trail (motility assay) (***, *P* = 0.0002). (I) Percentage of RH and hook-KO parasites that have attached to host cells (**, *P* = 0.0011). For panels E to I, the data are presented as means ± the SEM (*n* = three independent experiments; Student *t* test). (J) Survival curve of mice infected intraperitoneally with RH or hook-KO parasites. Log rank Mantel-Cox test: ***, *P* < 0.001 (*n* = 13 mice/parasite strain combined from two independent experiments).

To determine the localization of TgHOOK, transgenic parasites expressing a C-terminally HA-tagged HOOK protein (HOOK-HA) were generated by recombination at the endogenous locus. Western blot (WB) analysis revealed that HOOK-HA migrates at the predicted molecular weight of 79 kDa (Fig. 1A). Immunofluorescence assays (IFA) showed two distinct subcellular localizations, a punctate vesicular-like pattern preferentially enriched at the apical region of the parasite, similar to the distribution pattern of micronemes, and a concentrated signal at the conoid (Fig. 1B). Along these lines, the profile of *HOOK* mRNA deduced from the global cell cycle transcriptome (40) is comparable to the expression profile of genes coding for microneme proteins (see Fig. S1B). However, colocalization experiments indicated that TgHOOK staining does not coincide with micronemes, although TgHOOK-positive dotty staining could be found near these organelles (Fig. 1C). In addition, TgHOOK colocalized only partially with Rab11A, mostly in vesicles located at the basal pole of the parasite (see Fig. S1C). Thus, TgHOOK appears to define a novel vesicular compartment that may transiently interact with other parasite organelles.

### TgHOOK contributes to parasite motility, host cell attachment, invasion, and egress.

The role of TgHOOK was addressed through the generation of knockout (hook-KO) and inducible-knockdown (hook-iKD) strains. CRISPR/Cas9 technology was used to disrupt the gene in the RH strain leading to a frameshift mutation and a premature stop codon (41) (see Fig. S2A). The anhydrotetracycline (ATc) repressible system was used to generate the hook-iKD strain by promoter replacement (see Fig. S2B). Both strains were confirmed by genomic PCR analysis (see Fig. S2C). ATc-inducible repression of TgHOOK expression in the hook-iKD strain was also confirmed by WB analysis (see Fig. S3A). Parasites lacking TgHOOK exhibited a severe defect in lysis plaque formation (Fig. 1D and Fig. S3B and C). Phenotypic dissection of hook-KO parasites revealed a mild defect in intracellular growth when measured at 30 h postinvasion (Fig. 1E). The absence of TgHOOK also resulted in moderate defects in host cell invasion, induced egress, parasite motility and host cell attachment (Fig. 1F to I; see also Fig. S3D and F). The combined alterations in several steps of the lytic cycle correlated with a significant decrease in the virulence of hook-KO and hook-iKD parasites in mice (Fig. 1J; see also Fig. S3G).

10.1128/mbio.00458-23.2 FIG S2 (A) Schematic diagram of the strategy used to generate the hook-KO strain and the DNA/protein sequences resulting from a CRISPR-induced frameshift. The frameshift led to a premature stop codon. The sequence targeted by the guide RNA is highlighted in orange. (B) Schematic diagram of the strategy used to generate the hook-iKD strain. (C) PCR showing correct integration of the Tet07-inducible promoter. Download FIG S2, TIF file, 0.5 MB. Copyright © 2023 Dubois et al. 2023 Dubois et al. https://creativecommons.org/licenses/by/4.0/ This content is distributed under the terms of the Creative Commons Attribution 4.0 International license.

10.1128/mbio.00458-23.3 FIG S3 (A) The level of TgHOOK protein expression was assessed by WB in hook-iKD parasites. After 48h +ATc the protein was no longer detectable. Catalase was used as a loading control. (B) A plaque assay performed over 7 days revealed that, in the presence of ATc, hook-iKD parasites form smaller plaques compared to untreated parasites. (C) Quantification of lysis plaque area after 7 days of growth of hook-iKD parasites treated or not with ATc. (D) Invasion assay revealed a moderate defect in invasion capacity of hook-iKD parasites treated with ATc for 48 h compared to untreated parasites or the parental RHΔKu80Tati strain treated with ATc (*, *P* = 0.0123). (E) Hook-iKD +ATc (48 h) parasites display a significant defect in their motility compared to untreated parasites and ATc treated RHΔKu80Tati parasites (**, *P* = 0.0025). (F) Attachment assay revealed a significant attachment defect for hook-KO parasites compared to the parental control strain. Hook-iKD parasites in absence of ATc attached better than control parasites but revealed no defect when TgHOOK was depleted upon ATc treatment for 48 and 72 h. The data are expressed as the log_2_ fold change compared to the parental strain. (C to E) Data are presented as means ± the SEM (*n*= three independent experiments; Student *t* test). (G) Percentage of survival of mice infected with RHΔKu80Tati parasites treated with ATc and hook-iKD parasites treated or not with ATc. Mice were infected with 250 parasites intraperitoneally (log-rank Mantel-Cox: **, *P* = 0.001). (H) IFA performed in RH and hook-KO parasites, indicating no visible alteration of microneme (MIC2/MIC3), rhoptry (ARO), or dense granule (GRA3) biogenesis and localization. Scale bars, 5 μm. (I) IFA showing no defect in the localization of Rab5- and Rab7-positive endosome-like compartments in hook-KO parasites compared to control RH parasites. Rab5 and Rab7 proteins show a typical localization near the *trans*-Golgi network stained with the TgSortilin protein. Scale bars, 2 μm. Download FIG S3, TIF file, 7.1 MB. Copyright © 2023 Dubois et al. 2023 Dubois et al. https://creativecommons.org/licenses/by/4.0/ This content is distributed under the terms of the Creative Commons Attribution 4.0 International license.

### TgHOOK participates in the positioning and secretion of the micronemes and the discharge of the rhoptries.

**FIG 2:**
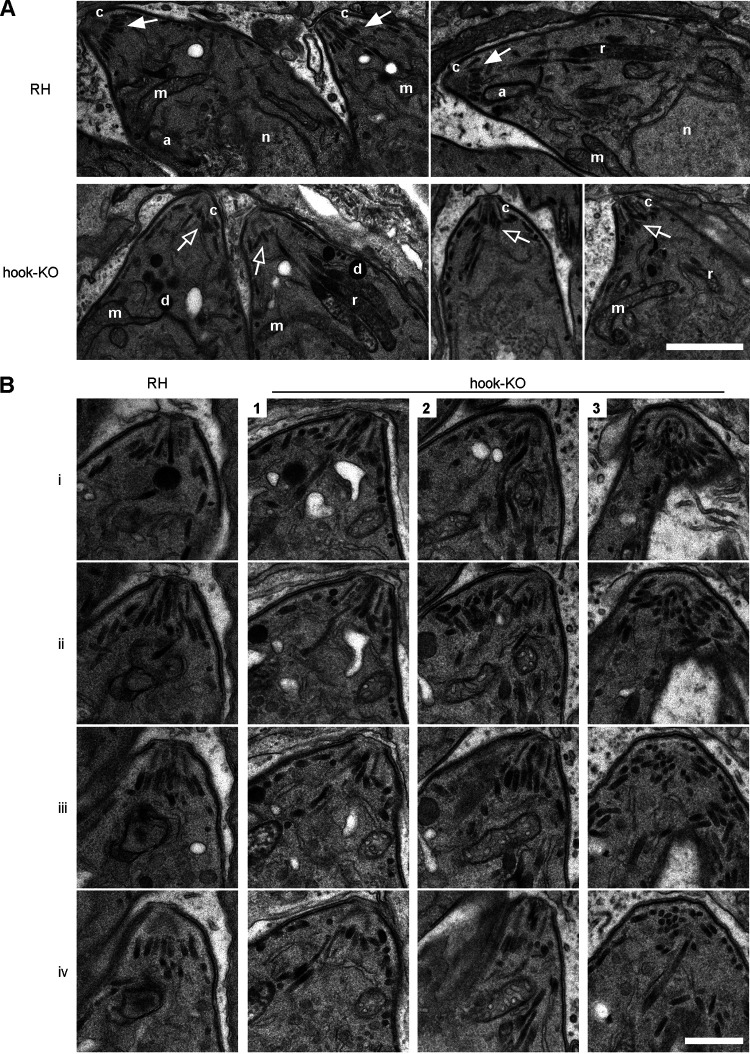
Morphological and ultrastructural comparison of RH and hook-KO by light and electron microscopy. (A) Electron microscopy analysis of the ultrastructure of RH parasites compared to hook-KO parasites inside distinct parasitophorous vacuoles (PVMs) reveals apical micronemes organized into a collar below the conoid (c) in RH control cells (white arrows) compared to hook-KO parasites which display disorganized apical micronemes (empty arrows) not aligned below the APR. Other organelle localization and structures are not affected, e.g., rhoptries (r), apicoplast (a), mitochondrion (m), nucleus (n), and dense granules (d). Scale bar, 1 μm. (B) Gallery of the apical part of the RH control parasite across four consecutive sections (i to iv) compared to three different hook-KO parasites from distinct PVMs (PVMs 1 to 3). Scale bar, 0.5 μm.

To dissect further TgHOOK depletion defect in motility and invasion, we scrutinized the positioning and morphology of the secretory organelles by IFA and electron microscopy (EM). By IFA, no apparent defect in microneme and rhoptry composition and localization was detected (see Fig. S3H). However, more detailed analysis by electron microscopy revealed that, in the absence of TgHOOK, the micronemes that accumulate below the APR were disorganized and failed to form a regular collar typically observed in RH control parasites (Fig. 2). This result indicates that TgHOOK contributes to the apical positioning of micronemes below the APR at the apical tip of replicating parasites. Collectively, the partial defect in egress, motility, and host cell attachment coupled to the alteration of apical microneme positioning suggest an impairment in microneme secretion.

**FIG 3:**
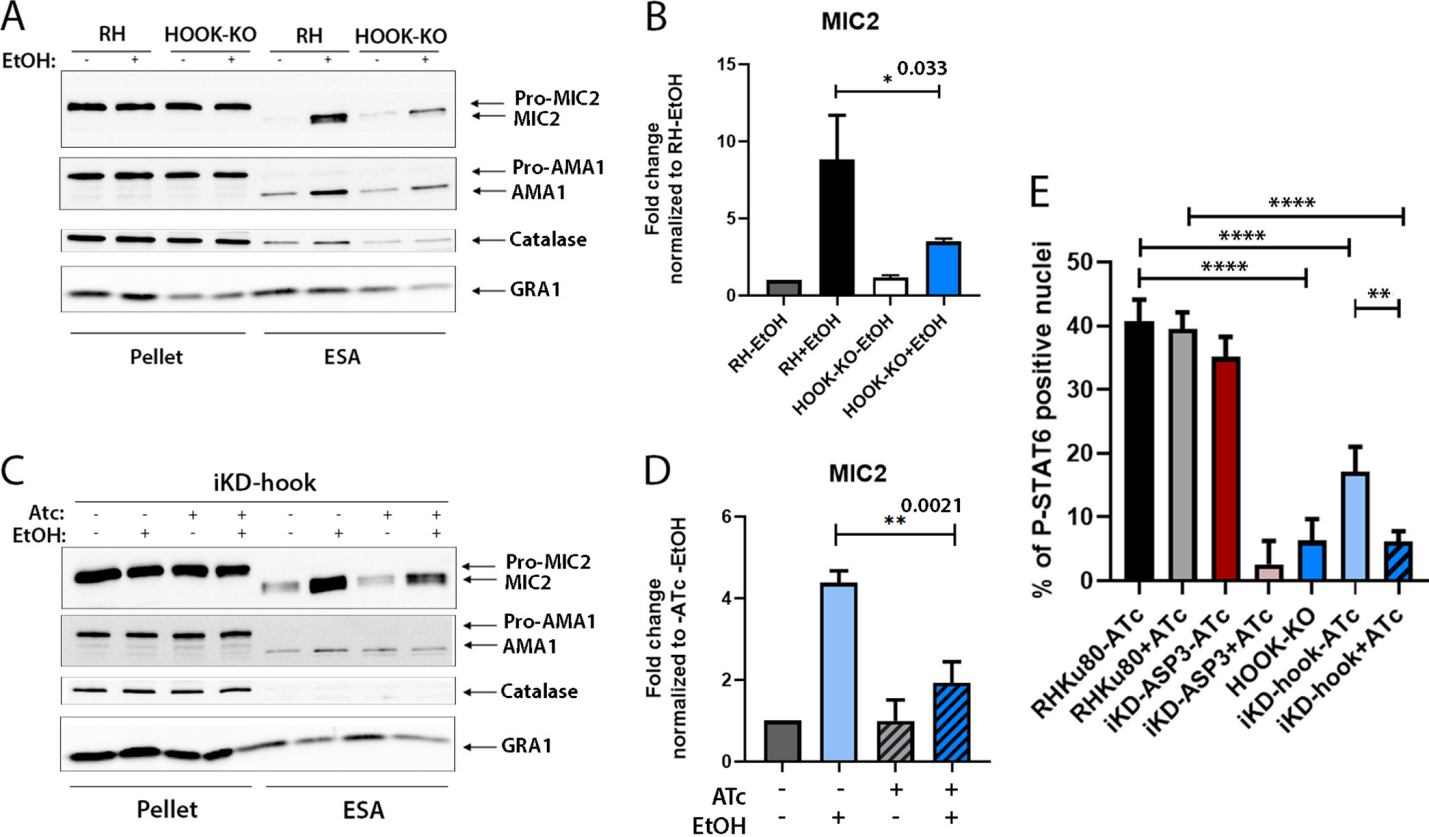
TgHOOK contributes to microneme and rhoptry secretion. (A and C) WB analyses showing a significant defect in MIC2 protein secretion in hook-KO parasites compared to the parental RH line (A) and in iKD-hook parasites treated with ATc for 70 h (+Atc) compared to iKD-hook nontreated parasites (–ATc) (C) when stimulated with 2% EtOH for 30 min. ESA, extracellular secreted antigens. Catalase was used as loading control. GRA1, dense granule protein 1. (B and D) Quantifications of MIC2 proteins detected in the ESA fraction of hook-KO strain ± EtOH, normalized to the RH –EtOH control condition (B) and of the iKD-hook strain ± ATc ± EtOH, normalized to the iKD-hook –ATc –EtOH condition (D). Student *t* test (*n* = 3 independent experiments). (E) Rhoptry discharge assay. Graph showing the percentage of P-STAT6-positive nuclei in fibroblasts infected by the indicated parasite strains in presence or absence of ATc treatment (Student *t* test; *, *P* < 0.05; **, *P* < 0.01; ****, *P* < 0.0001, *n* = 3 experiments).

Assessment of induced microneme secretion in hook-KO parasites upon stimulation with 2% ethanol (EtOH), revealed a significant 60% decrease in MIC2 protein released in the extracellular secreted antigen (ESA) fraction (Fig. 3A and B). In addition, no change in microneme protein proteolytic processing was detected. Comparable defect in microneme secretion was observed in hook-iKD parasites (Fig. 3C and D). Importantly, both TgHOOK depleted parasite mutants showed a defect in rhoptry discharge (Fig. 3E) measured quantitatively by STAT6 phosphorylation assay as a readout of injection of ROP16 into the host cell (42). Of note, a significant defect in rhoptry discharge was also observed in hook-iKD parasites in the absence of ATc, presumably due an alteration of HOOK protein level under the Tet07-inducible promoter (see Fig. S2B). In contrast, no alteration in constitutive dense granule secretion was observed by IFA (see Fig. S3H) in hook-KO replicating parasites, suggesting that the identified interaction between Rab11A and TgHOOK is not involved in the regulation of this process. Although HOOK contributes to early and late endosome trafficking in other eukaryotes, the Rab5A- and Rab7-positive endosome-like compartments did not display any obvious morphological alterations in hook-KO parasites (see Fig. S3I).

### Identification of TgHOOK-associated proteins.

**FIG 4:**
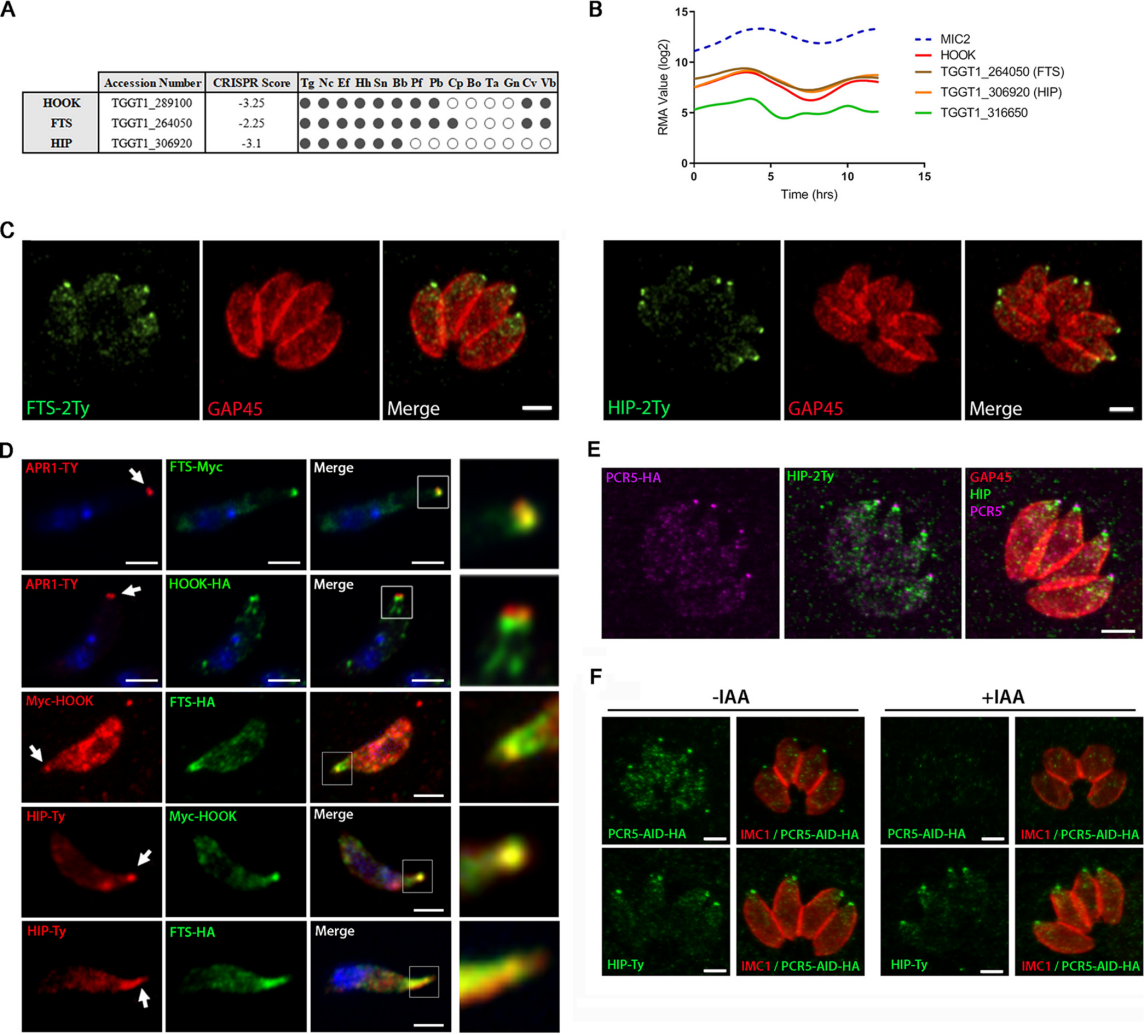
Characterization of TgFTS and TgHIP. (A) Table showing the conservation of *hook*, *Fts*, and *Hip* genes in Apicomplexa and Chromerida. The presence of orthologs is indicated by a black dot, and the absence by white dots. Organisms are indicated by abbreviations as follows: Toxoplasma gondii (Tg), Neospora caninum (Nc), *Eimeria falciformis* (Ef), *Hammondia hammondii* (Hh), *Sarcocystis neurona* (Sn), Besnoitia besnoiti (Bb), Plasmodium falciparum (Pf), Plasmodium berghei (Pb), Cryptosporidium parvum (Cp), Babesia bovis (Bo), *Theileria annulata* (Ta), *Gregarinicae niphandrodes* (Gn), Chromera velia (Cv), and Vitrella brassicaformis (Vb). An ortholog list was obtained using the EuPathDB.org transform-by-orthology feature. The CRISPR score obtained from a previous study (45). (B) Graph showing the transcriptional profiles of TgHOOK and candidate partners. T. gondii RH cell cycle microarray expression profiles of TgHOOK-associated proteins in intracellular replicating parasites were synchronized by using thymidine (40). (C) IFA experiments showing the localization of TgFTS and TgHIP endogenously tagged with 2Ty tag epitopes predominantly at the apical tip of the parasites but also detected in cytoplasmic vesicles enriched at the apical region. GAP45 was used as a pellicle marker to stain the parasite periphery. Scale bars, 2 μm. (D) IFA performed on extracellular motile parasites showed that HOOK-HA and FTS-Myc localize at the apical tip of the parasite, together with the apical ring marker Ty-APR1. Colocalization of TgFTS/TgHIP and TgHOOK was examined in the Myc-tagged hook-iKD line expressing endogenously HA-tagged FTS or Ty-tagged HIP. TgHOOK colocalizes with both proteins at the tip of the extruded conoid. TgFTS and TgHIP colocalize along and at the tip of the extruded conoid. (E and F) IFA showing the localization of HIP-2TY and PCR5-HA (E) in the PCR5-AID-HA/HIP-2Ty parasite strain and in parasites expressing (–IAA) or depleted (+IAA) for PRC5-AID-HA (F). Scale bars, 2 μm.

Proteins implicated in vesicular trafficking can dynamically assemble into complexes to achieve their function. To dissect further the role of TgHOOK, we performed coimmunoprecipitation (co-IP) of HOOK-HA, followed by mass spectrometry (MS) analysis to identify putative interacting partners (Table 1; see also Table S2). Three proteins were reproducibly detected by MS, including TGGT1_264050 that appears to be an ortholog of Fused Toes (FTS), a member of the FTS/HOOK/FHIP complex in model organisms, which belongs to the ubiquitin-conjugating enzyme subfamily of proteins (31, 33, 34). TGGT1_264050 was therefore named T. gondii FTS (TgFTS). Consistent with the conserved eukaryotic homologs, both TgHOOK and TgFTS were found conserved among most apicomplexans (Fig. 4A). In addition, TGGT1_306920 is a hypothetical protein and a novel member of this complex that was found only in the coccidian subgroup of the phylum and named here hook-interacting protein (TgHIP) (Fig. 4A). Of relevance, both TgFTS and TgHIP share a similar “microneme-like” cell cycle transcriptional profile with TgHOOK (Fig. 4B). In contrast, TGGT1_316650 does not share the expected “microneme-like” transcriptional profile and has been reported by the global mapping of protein subcellular location, Hyper LOPIT to be distributed across numerous subcellular fractions; 19S proteasome, cytosol, nucleus, and outlier (43). This protein has been frequently found in unrelated immunoprecipitations and hence was considered to be nonspecific and not prioritized for further characterization. To confirm the result obtained by mass spectrometry, TgFTS was endogenously tagged in the hook-iKD strain leading to N-terminally Myc-tagged HOOK and C-terminally hemagglutinin (HA)-tagged FTS. Immunoprecipitation assays with either anti-HA or anti-Myc antibodies confirmed that Myc-HOOK interacts with FTS-HA (see Fig. S4A).

**TABLE 1:** Number of unique peptides of TgHOOK, TgFTS, and TgHIP identified by MS after HOOK-HA protein immunoprecipitation^*a*^

Protein	Accession no.	Peptide count		
		Expt 1	Expt 2	Expt 3
TgHOOK	TGGT1_289100	40	44	30
HOOK interacting protein (TgHIP)	TGGT1_306920	15	8	11
Fused Toes (TgFTS)	TGGT1_264050	8	7	0
Hypothetical	TGGT1_316650	24	31	2

a The results from three independent experiments are shown.

10.1128/mbio.00458-23.9 TABLE S2 Summary of the MS results in Table 1. MS was performed on coimmunoprecipitated material (from HOOK-HA parasite lysate), and the total number of identified peptides was quantified (*n* = 3 independent experiments). Download Table S2, XLSX file, 0.02 MB. Copyright © 2023 Dubois et al. 2023 Dubois et al. https://creativecommons.org/licenses/by/4.0/ This content is distributed under the terms of the Creative Commons Attribution 4.0 International license.

Next, to determine TgFTS and TgHIP localization, both proteins were C-terminally epitope-tagged by homologous recombination at their respective endogenous loci. IFA showed that FTS-2Ty and HIP-2Ty predominantly localized at the parasite apical tip of intracellular replicating parasites, although dotty cytoplasmic staining for these tagged proteins, and likely corresponding to vesicles, could be detected (Fig. 4C). Deletion of TgHOOK correlates with defects in extracellular parasite functions. Thus, we next assessed the colocalization of TgFTS, TgHIP and TgHOOK in extracellular parasites that have been allowed to move for 10 min on poly-l-lysine-coated coverslips prior fixation. This experiment allowed to assess protein localization at the apical tip of parasites displaying an extruded conoid. First, after N-terminal Ty-tagging of the apical polar ring marker APR1 (see Fig. S4B), we observed that in parasites without an extruded conoid, FTS-Myc colocalized with APR1-Ty. HOOK-HA only partially colocalized with APR1, being also detected slightly beneath this APR marker (Fig. 4D). HIP-Ty and FTS-HA colocalized along and at the tip of the extruded conoid (Fig. 4D). We also found a colocalization between Myc-HOOK and HIP-Ty or FTS-HA at the tip of extruded conoids (Fig. 4D). The three components of the complex failed to be detected by ultrastructure expansion microscopy, thus hampering higher resolution of their localization at the conoid. As an alternative, we epitope-tagged TgHIP in the inducible mutant of Pcr5 (Pcr5-mAID-HA/HIP-2Ty), a marker of the PCRs (44). Colocalization experiments clearly showed that TgHIP is below the PCRs, suggesting that the T. gondii HOOK-FTS-HIP complex localizes at the conoid cone (Fig. 4E). Furthermore, destabilization of the PCRs by depletion of Pcr5 did not impact on TgHIP localization at the apical tip of intracellular parasites, confirming that this protein does not associate with this structure (Fig. 4F).

### TgFTS and TgHIP critically contribute to microneme and rhoptry secretion.

**FIG 5:**
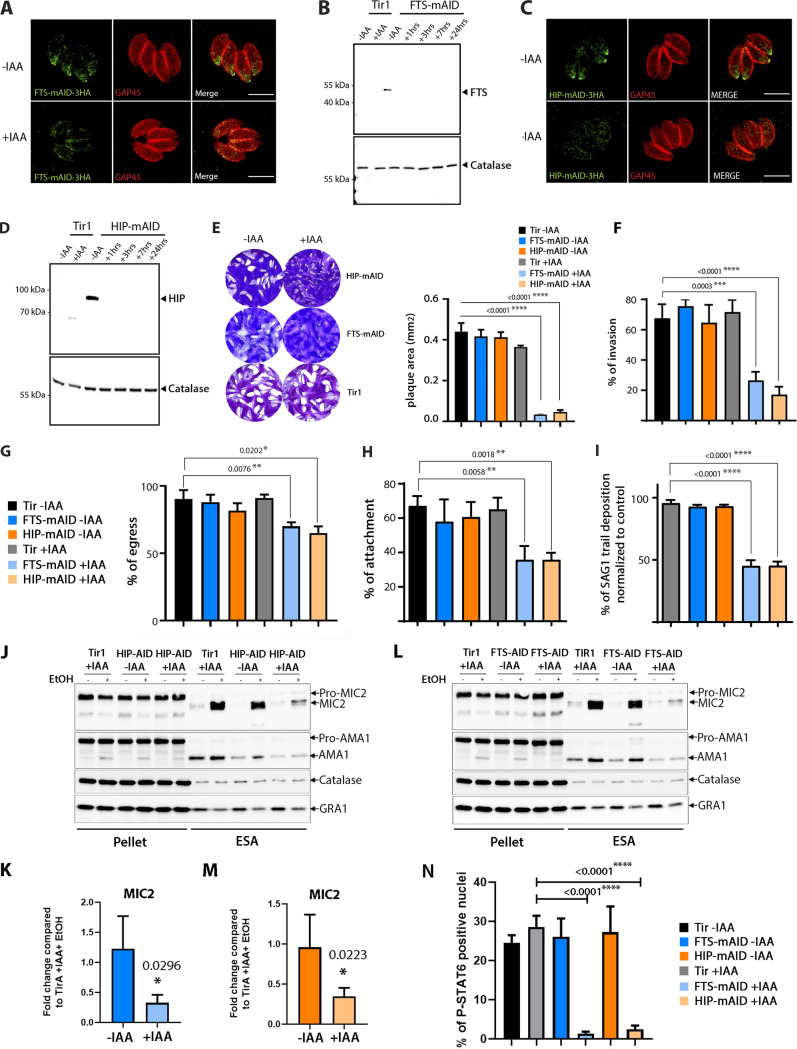
TgFTS and TgHIP critically contribute to host cell invasion and are required for optimal microneme secretion. (A to D) FTS-mAID-HA and HIP-mAID-HA are regulated by IAA. (A to C) IFA showing the localization of FTS-mAID-HA and HIP-mAID in intracellular parasites in the absence of IAA (–IAA). Treatment with IAA for 24 h leads to TgFTS and TgHIP degradation. GAP45 was used to stain the parasite periphery. Scale bar, 5 μm. (B to D) Western blot analysis showing the depletion of TgFTS and TgHIP proteins after 1 h treatment with IAA. Catalase was used as a loading control. (E) Plaque assay performed over 7 days in the presence or absence of IAA. FTS-mAID-HA and HIP-mAID-HA depleted parasites (+IAA) demonstrate a severe lytic cycle defect (quantification of lysis plaque areas [mm^2^]) compared to the parental control (Tir1) and to untreated conditions (–IAA). for the indicated parasites strains treated or not with IAA. (F) TgFTS- and TgHIP-depleted parasites (+IAA) are severely impaired in host cell invasion compared to the parental control (Tir1 +IAA) and to untreated conditions (–IAA). FTS, ***, *P* = 0.0003; HIP, ****, *P* ≤ 0001. (G) EtOH-induced parasite egress was modestly affected in the absence of HIP and FTS. (H and I) FTS-mAID-HA and HIP-mAID-HA parasites treated with IAA for 24-h display defects in host cell attachment (FTS, **, *P* = 0.0058; HIP, **, *P* = 0.0018) (H) and motility (FTS and HIP, ****, *P* < 0.0001) assessed by the trail deposit assay (I). For panels E to I, the data are presented as means ± the SEM (*n* = three independent experiments; Student *t* test). (J and L) Western blot showing that TgFTS- and TgHIP-depleted parasites (+IAA) were impaired in microneme secretion when stimulated with 2% EtOH for 30 min compared to control conditions. Pellet, parasite pellet fraction; ESA, extracellular secreted antigen fraction; catalase, loading control for parasite number and integrity. The constitutive secretion of the dense granule protein GRA1 was unaffected. (K and M) Graphs indicate the quantifications of MIC2 detected in the ESA fractions of ±IAA-treated parasites, normalized to the Tir1 (+IAA, +EtOH) control condition. Data presented as means ± the SEM (FTS, *, *P* = 0.0296; HIP, *, *P* = 0.0223; Student *t* test, *n* = 3 independent experiments). (N) Rhoptry discharge assay. Graph showing the percentage of P-STAT6-positive nuclei in fibroblasts infected by the indicated parasite strains. (****, *P* < 0.0001, *n* = 3 experiments).

TgHOOK, TgFTS, and TgHIP form a complex at the conoid and presumable function together to ensure motility, invasion, and egress. To decipher specifically the role of TgFTS and TgHIP, the fast protein destabilization system based on the auxin-induced degron (AID) was applied (45, 46). A C-terminal miniAID (mAID) tag was added to the endogenous locus of TgFTS and TgHIP in a transport inhibitor response 1 (Tir1)-expressing strain. In this strain, mAID-fused proteins are efficiently targeted for proteasomal degradation upon the addition of indole-3-acetic acid (IAA). FTS-mAID-HA and HIP-mAID-HA parasites clones were confirmed by genomic PCR (see Fig. S5A) and IFA. The mAID-fused proteins showed the same subcellular localization as the endogenously tagged FTS-Myc/HA and HIP-Ty (Fig. 5A to D) with FTS-mAID migrating at the expected size of 46 kDa. In contrast HIP-mAID was migrating at ~85 kDa instead of 61 kDa, an unexplained aberrant migration which we also observed for the HIP-2Ty protein (Fig. 5B and D). Upon IAA treatment for 1 h, both FTS-mAID and HIP-mAID became undetectable by Western blot (Fig. 5B and D). Depletion of either TgFTS or TgHIP strongly alters parasite fitness as shown by plaque assays performed over 7 days in the presence of IAA, compared to the parental (Tir1) strain (Fig. 5E). The phenotypic consequences of TgFTS or TgHIP depletion revealed a severe defect in host cell invasion (Fig. 5F). Both mutants exhibited normal intracellular growth (see Fig. S5B and C), as well as conoid extrusion (see Fig. S5D) and a moderate but significant decrease in egress (Fig. 5G). Consistent with the defects observed in parasite motility and host cell attachment (Fig. 5H and I), both mutants were similarly impaired (60 to 70% decrease compared to the “–IAA” condition) in induced microneme secretion in the presence of 2% EtOH (Fig. 5J to M). A severe defect in rhoptry discharge for both FTS and HIP deficient parasites was also observed (Fig. 5N). Importantly, secretory organelle positioning and morphology as visualized by IFA and EM, respectively, were not affected after 24 h of IAA treatment (see Fig. S5E and F; see also Fig. S6A and B). Most notably, the micronemes appeared properly arranged into a regular collar at the vicinity of the APR contrasting with the alteration observed in hook-KO parasites (see Fig. S6).

10.1128/mbio.00458-23.4 FIG S4 (A) Coimmunoprecipitation assays performed on lysates from the Myc-tagged hook-iKD parasites expressing endogenously HA-tagged FTS. The hook-iKD strain was used as a negative control. Western blot (WB) analysis indicated that Myc-HOOK was immunoprecipitated with FTS-HA (IP anti-HA) and, reversely, that FTS-HA was immunoprecipitated with Myc-HOOK (IP anti-Myc). SN, supernatant (input); FL, flowthrough; IP, bead fraction. IgG (immunoglobulins). (B) WB analysis showing the expression of FTS-Myc and APR1-2Ty (at the expected size of 52 kDa) in FTS-Myc/APR1-2Ty-expressing parasites (up) and of HOOK-HA and APR1-2Ty in HOOK-HA/APR1-2Ty parasites (down). Download FIG S4, TIF file, 0.5 MB. Copyright © 2023 Dubois et al. 2023 Dubois et al. https://creativecommons.org/licenses/by/4.0/ This content is distributed under the terms of the Creative Commons Attribution 4.0 International license.

10.1128/mbio.00458-23.5 FIG S5 (A) Integration PCR, with a schematic diagram showing PCR primer pairs spanning HXGPRT resistance cassette and the 3′UTR outside of homology region, used to verify the correct integration. (B to D) Quantification of the percentage of vacuoles containing 2, 4, 8, 16, or 32 parasites after 30 h of parasite growth in HFF (B and C) and of parasites displaying a protruded conoid (D) for the parental (TirA) and FTS/HIP-mAID lines treated or not with IAA for 24 h. Parasite replication and conoid protrusion were unaffected in the absence of TgFTS and TgHIP. The data are presented as means ± the SEM from three independent biological experiments. (E and F) Secretory organelles visualized by IFA in FTS/HIP-mAID-HA parasites treated or not with IAA using anti-MIC2 to stain the micronemes and anti-ARO to stain the rhoptries. Actin was used as a marker of the cytoplasm and nucleus, GAP45 stains the pellicle. Scale bars, 5 μm. Download FIG S5, TIF file, 1.4 MB. Copyright © 2023 Dubois et al. 2023 Dubois et al. https://creativecommons.org/licenses/by/4.0/ This content is distributed under the terms of the Creative Commons Attribution 4.0 International license.

10.1128/mbio.00458-23.6 FIG S6 Gallery of electron micrographs from six consecutive sections (i to vi) through the apical part of two different cells (1 and 2) from both HIP-mAID-HA (A) and FTS/HIP-mAID-HA (B) treated with IAA for 24 h demonstrating no obvious morphological defects in the arrangement of micronemes and other apical organelles, e.g., rhoptries (r), apicoplast (a), mitochondrion (m), nucleus (n), and dense granules (d). Scale bars, 1 μm. Download FIG S6, TIF file, 6.5 MB. Copyright © 2023 Dubois et al. 2023 Dubois et al. https://creativecommons.org/licenses/by/4.0/ This content is distributed under the terms of the Creative Commons Attribution 4.0 International license.

Taken together, depletion of either TgFTS or TgHIP exhibit comparable phenotypes suggesting that both proteins contribute toward the same biological process, whereas deletion of TgHOOK results in a broader array of milder phenotypes, including defects in intracellular growth, parasite egress, and in apical microneme positioning.

### Interactions between TgHOOK, TgFTS, and TgHIP.

To decipher the interactions between the members of the T. gondii HOOK-FTS-HIP complex, TgHOOK was endogenously Ty-tagged in the HIP-mAID-HA (HIP-mAID-HA/HOOK-Ty) (see Fig. S7A and B) and coimmunoprecipitations coupled to MS were performed. The pulldown of HIP-HA in the absence of IAA revealed an interaction with HOOK-Ty; however, no TgFTS peptides were detectable by MS in the IP (Table 2; see also Table S3 and Fig. S7C and D). In contrast, HOOK-Ty pulldown in the absence of IAA reproduced the initial results with the entirety of the complex represented in the immunoprecipitation (Table 2; see also Table S3 and Fig. S7C and D). Analysis of the HOOK-Ty pulldown in the presence of IAA, resulting in the depletion of HIP-HA, indicated that even in the absence of HIP-HA an unaltered HOOK-FTS interaction is present (Table 2; see also Table S3). Concordantly, FTS-HA pulldown confirmed the existence of the HOOK-FTS-HIP complex (Table 2; see also Table S3). These results indicate that TgHOOK interacts with both TgHIP and TgFTS and that TgFTS is not required for TgHIP interaction with TgHOOK. Our results also point to TgFTS not directly interacting with TgHIP.

**TABLE 2:** Abridged results of IP-MS analysis^*a*^

Expt	Strain	Protein	Peptide count	
			−IAA	+IAA
Pulldown HIP	HIP-mAID-HA (HOOK-Ty)	HOOK	12	NA
	HIP-mAID-HA (HOOK-Ty)	FTS	0	NA
	HIP-mAID-HA (HOOK-Ty)	HIP	5	NA
Pulldown HOOK	HOOK-Ty (HIP-mAID-HA)	HOOK	45	41
	HOOK-Ty (HIP-mAID-HA)	FTS	7	7
	HOOK-Ty (HIP-mAID-HA)	HIP	4	0
Pulldown FTS	FTS-mAID-HA	HOOK	39	NA
	FTS-mAID-HA	FTS	9	NA
	FTS-mAID-HA	HIP	8	NA

a Mass spectrometry was performed on coimmunoprecipitated material (±IAA 24 h) and the total number of identified peptides was quantified. NA, Non applicable.

10.1128/mbio.00458-23.7 FIG S7 Deciphering the interactions between the members of the HOOK-FTS-HIP complex. (A) Western blot showing the expression of TgHIP and TgHOOK proteins in the HIP-mAID strain stably expressing HOOK-2Ty treated or untreated with IAA for 1 h. Catalase was used as a loading control. (B) IFA performed in extracellular adherent parasites showing the colocalization of TgHIP and TgHOOK at the apical tip of HIP-mAID/HOOK-2Ty parasites. Scale bar, 5 μm. (C) Western blot of TgHIP pulldown with TgHOOK co-IP. (D) Western blot of TgHOOK pulldown with TgHIP co-IP. Download FIG S7, TIF file, 0.8 MB. Copyright © 2023 Dubois et al. 2023 Dubois et al. https://creativecommons.org/licenses/by/4.0/ This content is distributed under the terms of the Creative Commons Attribution 4.0 International license.

10.1128/mbio.00458-23.10 TABLE S3 Summary of the MS results in Table 2. MS was performed on coimmunoprecipitated material (±IAA 24 h), and the total number of identified peptides was quantified. Download Table S3, XLSX file, 0.02 MB. Copyright © 2023 Dubois et al. 2023 Dubois et al. https://creativecommons.org/licenses/by/4.0/ This content is distributed under the terms of the Creative Commons Attribution 4.0 International license.

**FIG 6:**
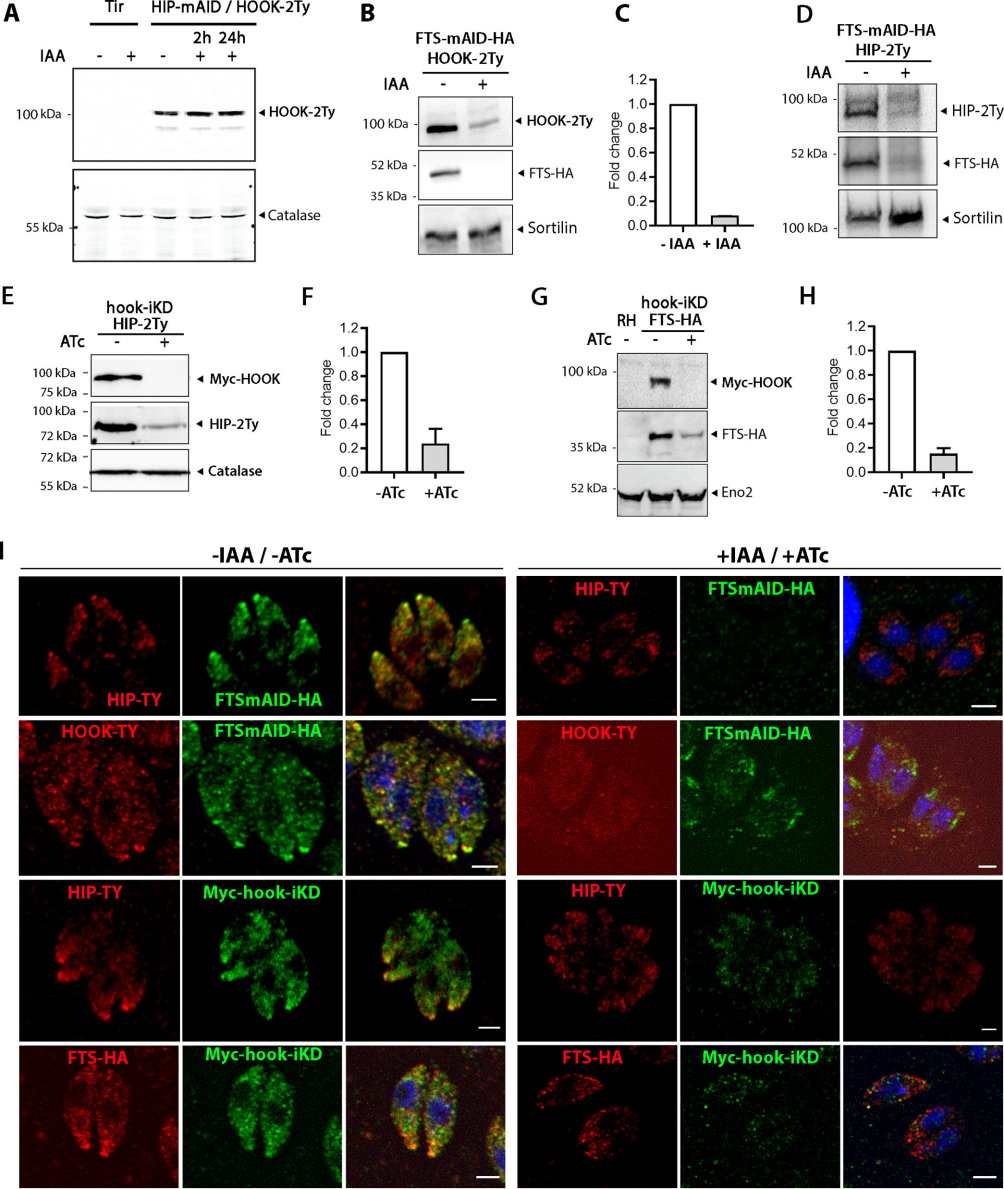
Interactions between TgHOOK, TgFTS, and TgHIP. (A) WB analysis showing that HOOK-2Ty abundance is unaffected by the degradation of HIP-AID-HA (treated with IAA for 2 and 24 h). Catalase was used as a loading control. (B and C) WB (B) and quantification (C) showing that HOOK-2Ty abundance is reduced upon downregulation of TgFTS protein in FTS-AID-HA/HOOK-2Ty parasites treated with IAA (24 h) compared to untreated conditions (–IAA) (*n* = three independent experiments). Sortilin was used as a loading control. (D) WB showing that HIP-2Ty protein level is reduced upon downregulation of TgFTS in FTS-AID-HA/HIP-2Ty parasites treated with IAA (24 h) compared to untreated conditions (–IAA). Catalase was used as a loading control. (E to G) WB showing that FTS-HA and HIP-2Ty abundance is reduced upon downregulation of Myc-HOOK protein in hook-iKD/FTS-HA or HIP-2Ty parasites induced with ATc (48 h) compared to untreated conditions (–ATc). Enolase 2 (Eno2) and catalase were used as a loading control. (F to H) Graphs indicate the quantification of FTS-HA and HIP-2Ty protein levels in hook-iKD parasites treated for 48 h compared to untreated parasites (*n* = three independent experiments). (I) IFA showing the localization of TgHOOK, TgFTS, and TgHIP in the presence (–IAA/–ATc) or in the absence of one of the other members of the complex (+IAA, +ATc) as indicated. Depletion of TgFTS (FTSmAID-HA + IAA) leads to reduced amount of TgHOOK and TgHIP and depletion of HOOK (Myc-hook-iKD + ATc) leads to reduced levels of TgFTS and TgHIP. Scale bar, 2 μm.

Typically, withdrawing one component of a complex, can destabilize, or mistarget the other components of the complex. To further assess the role played by each protein in complex stability, we generated an additional parasite line, in which TgHOOK was endogenously Ty-epitope tagged in the FTS-mAID-HA (FTS-mAID-HA/HOOK-2Ty). We observed that HOOK-2Ty protein expression was unaffected upon TgHIP depletion (Fig. 6A). In contrast, depletion of TgFTS upon IAA treatment resulted in a drastic decrease in both HOOK-2Ty and HIP-2Ty protein expression (Fig. 6B to D). Similarly, HOOK depletion in iKD-hook/FTS-HA or iKD-hook/HIP-TY strains reproducibly led to a strong reduction in TgFTS and TgHIP abundance suggesting that complex formation is essential to stabilize its components (Fig. 6E to H). These results obtained by WB analysis were further confirmed by IFA (Fig. 6I). In parasites depleted for TgHOOK, only a faint detection of TgFTS and TgHIP could be observed at the apical tip or in the cytosol of HOOK-depleted parasites. Similarly, depletion of TgFTS drastically reduced TgHIP and TgHOOK signal compared to untreated control parasites.

## DISCUSSION

Studies on T. gondii have highlighted the functional repurposing of conserved endosomal trafficking proteins toward the biogenesis of specialized apicomplexan secretory organelles during parasite replication (47). Consequently, endosomal transport regulators constitute prime candidates to contribute to the ultimate step of apical replenishment and exocytosis of organelles in motile and invasive parasites. In eukaryotic models, the conserved FHF complex composed of FTS, FHIP, and HOOK has been found to promote early and late endosomal vesicle transport and fusion (31, 32, 34, 35). In T. gondii, homologs of HOOK and FTS are also associated but an ortholog of FHIP is lacking. Instead, a coccidian specific HOOK interacting partner (HIP) has been identified that might fulfill a similar role. Conspicuously we observed several low complexity regions within TgHIP, but no clear features or domains which would specify how it may achieve its molecular function. The three proteins of the complex colocalize at the apical tip of the parasite likely associated with the cone made of tubulin fibers. In contrast to TgFTS and TgHIP, TgHOOK localization exhibits an abundant vesicle-like pattern also detected at the basal pole of the parasite, where it partially colocalizes with Rab11A. In addition, TgHOOK-deficient parasites are modestly perturbed in all steps of the lytic cycle, including parasite replication. Presumably TgHOOK regulates distinct trafficking processes at different steps of the cell cycle. This implies that TgHOOK could regulate the trafficking of intracellular compartments other than micronemes in a TgHIP- and TgFTS-independent manner. Although a role in early and late endosomal trafficking has been described for HOOK in other eukaryotes, no obvious alterations in Rab5- and Rab7-positive endosomal compartments could be observed in hook-KO parasites, consistent with the lack of interactions observed by pulldown between HOOK-HA and Rab5 or Rab7. In contrast, Rab11A was identified as a preferential partner of HOOK-HA suggesting that TgHOOK may regulate Rab11A-dependent trafficking pathways, notably for parasite replication, an aspect that requires further investigation.

In support of TgHOOK possible role in several trafficking pathways and thus in multiple processes, deletion of TgHOOK led to a broad array of partial defects in parasite replication, host cell attachment and invasion, parasite motility, and egress. This array of phenotypes was similarly observed in the ATc repressible and in the hook-KO strains, suggesting that the partial phenotypes are not explained by incomplete depletion. Of note, hook-iKD parasites treated with ATc attached better and displayed a defect in rhoptry discharge compared to the parental line in the absence of ATc (Fig. 3E; see also Fig. S3E). This might be the result of an altered level of TgHOOK expression in hook-iKD parasites compared to endogenous expression. TgHOOK-depleted parasites display an altered positioning of micronemes, which fail to arrange in a regular collar at the proximity of the APR. This defect was not observed for TgFTS and TgHIP depleted parasites, pointing to a specific role for TgHOOK in microneme anchoring at the APR independently of TgFTS and TgHIP. This altered apical positioning of the microneme might affect the sustained organelle discharge necessary for egress, gliding, and invasion. Indeed, microneme secretion has been documented to occur in successive waves, the current hypothesis is that groups of micronemes are sequentially replenished and trafficked through the conoid to be secreted at the apical tip (2, 11, 12). Of relevance, TgDLC8a is a microtubule motor subunit localized within the conoid that has been recently reported to critically contribute to microneme secretion and that is considered to be an essential regulator of microneme apical replenishment (14, 15). Similarly, the HOOK/FTS/HIP complex described here appears to be implicated in the same process. However, the positioning of the micronemes is not altered in TgFTS- and TgHIP-depleted parasites and yet microneme exocytosis is more severely impacted. Curiously, TgHIP and TgFTS only resulted in a modest egress defect suggesting sufficient residual microneme secretion to ensure parasite egress, but possibly not enough replenishment for subsequent invasion. Noteworthy, a similar defect in microneme secretion for TgHOOK-depleted parasites has been also observed in a recent study (48).

Along these lines, previous studies on the depletion of proteins that critically participate in microneme exocytosis such as TgAPH (42, 49) or, more recently, TgGC and TgUGO (50) and resulted in a full block in microneme secretion assay, was translated in a complete block in egress. Here, the intermediate phenotypes observed for TgHOOK, TgFTS, and TgHIP do not show a perfect correlation between the two assays. This suggests that the EtOH-induced microneme secretion on purified parasites does not reflect the requirement for BIPPO induced egress of intracellular parasites. A comparison of the observed phenotypes points toward incompletely overlapping functions between TgHOOK and TgFTS-TgHIP.

Moreover, the interactions between members of the complex investigated by coimmunprecipitations indicate that TgHOOK is the central member binding to both TgHIP and TgFTS; however, there is no evidence that FTS-HIP are directly interacting. This is consistent with previous findings obtained in other eukaryotic systems showing that HOOK binds both FTS and FHIP via its C-terminus domain (35). In line with this, we also found that HOOK-FTS-HIP complex formation via FTS-HOOK interactions is required to stabilize the three proteins of the complex otherwise degraded. HOOK is known to bind both microtubule motors and cargos via its interaction with Rab GTPases (29–31). It is therefore plausible that TgHOOK interacts with microtubule motors, notably dynein, to position the micronemes at the vicinity of the APR. Upon induction of microneme secretion, TgFTS and TgHIP localize at the cone of the conoid and likely bind to TgHOOK to facilitate the transport of micronemes to the tip of the parasite to reach the plasma membrane where exocytosis takes place. This studying model is also in agreement with the localization of the three proteins of the complex along and at the tip of extruded conoid in extracellular parasites.

Of considerable relevance, TgHOOK- and, to a larger extent, TgFTS- and TgHIP-deficient parasites display an impairment in rhoptry discharge that is sufficient to explain their modest and severe invasion defect, respectively. Previously the microneme proteins MIC8 and more recently, TgCRMP proteins have been reported to critically participate in rhoptry discharge (51–53). It is therefore plausible that the microneme secretion defect prevents rhoptry discharge. However, since microneme secretion is only partially altered, we cannot exclude that the T. gondii HOOK/FTS/HIP complex directly contributes to the process of rhoptry protein secretion by a yet unknown mechanism. Taken together, this study has identified important trafficking regulators involved in the final steps of organelle discharge to ensure motility, invasion, and egress.

## MATERIALS AND METHODS

### Primers.

The primers used in this study are listed in Table 3.

**TABLE 3:** Primers used in this study

Primer description	Primer sequence (5′–3′)
Fw primer to screen iKO integration (primer 2, Fig. S1)	CCGGAATTCAAGAAAAAATGCCAACGAGTAGTTTTC
Fw primer to screen iKO integration (primer 3, Fig. S1)	TGGCCAGGGCGAATTGGGTACCGAGC
Fw primer for KI-AMA1-Myc-BirA	CGGGGTACCCAAGCCTGTAAAAGACAGAAGACGTCCTG
Rev primer for KI-AMA1-Myc-BirA	CGGCCTGCAGGGTAATCCCCCTCGACCATAACATGTG
Fw primer for GST-Rab11A	CGGggatccGAACAAAAACTCATCTCAGAAGAGGATCTGATGGCGGCTAAAGATGAATACTACG
Rev primer for GST-Rab11A	gcggccgcTCAGGCGGAACAGCAGCCAC
Fw primer annealing downstream of ATG of HXGPRT resistance cassette (primer 1, Fig. S3)	CGACAACACCTTCTACAACGCTG
Universal reverse primer for CRISPR/Cas9 guide	AACTTGACATCCCCATTTAC
Fw primer for KI-HOOK-HA-DHFR	tacttccaatccaatttagcGCAAAGATGACATGGCGAAGCAGATGATG
Rev primer for KI-HOOK-HA-DHFR	tcctccacttccaattttagcCGCCTCCCGAGGTGTGACAGAATC
Fw primer for KI-HOOK-3Ty-DHFR	GCGGGTACCGATGGAGGCTTTGAGCAAACACG
Rev primer for KI-HOOK-3Ty-DHFR	GCGATGCATGCGCCTCCCGAGGTGTGACAG
gRNA for iKO tet system, pSAG1::CAS9-GFP-U6::sgHOOK2	GAGTCCGCACCTCTACACGGGGTTTTAGAGCTAGAAATAGC
Fw primer for iKD Tet system	CTTCGTCAATAATGTTTCATCCATTTCGTTCATGTTTGCGGATCCGGGG
Rev primer for iKD Tet system	GTCGAGCAAGGGCGCGTGAGTGAAAGACATCAGGTCCTCCTCGGAGATGA
Fw primer to screen iKO integration (primer 1, Fig. S1)	GCGCCATGGTAACCGTTTAGCTGCAACAATCC
Rev primer to screen iKO integration (primer 4, Fig. S1)	GCGACTAGTAACTCGAGACCCATTTGAGAACC
gRNA for HOOK KO, pSAG1::CAS9-GFP-U6::sgHOOK1	GACCGAAATGGCGGCCCTGAGTTTTAGAGCTAGAAATAGC
Fw primer to screen HOOK-KO	CGTCCCAGACTCAAAGAAGAAATG
Rev primer to screen KO	GGTTCCAAGGCGAGTGCAGC
Fw primer for KI-FTS-cMyc-HXGPRT and KI-FTS-HA-DHFR	TACTTCCAATCCAATTTAGCATTCCGTCATGGGACAATCTTCGAG
Rev primer for KI-FTS-cMyc-HXGPRT and KI-FTS-HA-DHFR	TCCTCCACTTCCAATTTTAGCTTCGGCGTTGAAGAGGTTGGCGCC
gRNA for KI-APR1-2Ty, pSAG1::CAS9-GFP-U6::sgAPR1	ATTCACATCGGCCTATATACGTTTTAGAGCTAGAAATAGC
Fw primer for C terminus KI of APR1-Ty in KI-FTS-cMyc and KI-HOOK-HA	GGGACCCCTCCGCCGTGGAGAGTTAAAAGCGCTAGCAAGGGCTC GGG
Rev primer for C terminus KI of APR1-Ty in KI-FTS-cMyc and KI-HOOK-HA	CAAAAACTGATACCGAGTGTCGCACTGGCAATACGACTCACTATA GGG
gRNA for FTS-mAID-HA and KI-2Ty, pSAG1::CAS9-GFP-U6::sgFTS	GTGTAGCTTGCTGGTGGCCATGTTTTAGAGCTAGAAATAGC
Fw Primer, KOD PCR for FTS-mAID and KI-2Ty	AGTACTGGCGCCAACCTCTTCAACGCCGAAGCTAGCAAGGGCTCGGG
Rev primer, KOD PCR for FTS-mAID and KI-2Ty	AGAAAAGACTACCAGTAGAACCCGCAAAGCATACGACTCACTATAGGG
Rev primer screen integration FTS (primer 2, Fig. S3)	CGCACTCCCGGTACTATTTCC
gRNA for HIP-mAID-HA and KI-2Ty, pSAG1::CAS9-GFP-U6::sgHIP	GCCTCGGCGATGCCCCTTTGCGTTTTAGAGCTAGAAATAGC
Fw primer, KOD PCR for HIP-mAID and KI-2Ty	CCGTCCGCTTCGTCTTCAACAGCGTCTACGGCTAGCAAGGGCTCGGG
Rev primer, KOD PCR for HIP-mAID and KI-2Ty	ATTGACTGCAGGGCTGTAGAGAGCTACGCCATACGACTCACTATAGGG

### Cloning of DNA constructs.

All amplifications were performed with either KOD polymerase (Novagen) or Q5 polymerase (New England Biolabs). RNA was isolated using TRIzol extraction. Total cDNA was generated by reverse transcription-PCR using the Superscript II reverse transcriptase (Invitrogen) according to the manufacturer’s protocol. KI-AMA1-Myc-BirA vector was generated using the primer pair listed in Table 3 on gDNA and subsequently cloned into pT8-TgN21APH-MycBirA_HX between KpnI and NsiI restriction sites. KI-HOOK-HA vector was generated using the primer pair listed in Table 3 on gDNA and subsequently cloned into pLIC-HA-DHFR. KI-HOOK-3Ty vector was generated using the primer pair listed above in Table 3 on gDNA and subsequently cloned into pG152-KI-3Ty-lox-SAG1_3′UTR-HX-U1-lox between KpnI and NsiI restriction sites. KI-FTS-HA and KI-FTS-cMyc vectors were generated using the primer pair listed in Table 3 on gDNA and subsequently cloned into pLIC-HA-DHFR and pLIC-cMyc-HXGPRT, respectively. The gRNA/Cas9 vectors used in this study were made using a Q5 site-directed mutagenesis kit (New England Biolabs) with pSAG1::CAS9-GFP-U6::sgUPRT as a template (27).

The EuPaGDT tool was used for guide selection (http://grna.ctegd.uga.edu/).

### Parasite transfection and selection of stable transfectants.

T. gondii tachyzoites were transfected by electroporation as previously described (36). KI-AMA1-Myc-BirA strain was generated by transfection of RHΔKu80 (here referred as ΔKu80) (37, 38) with linearized KI-AMA1-Myc-BirA_HX. KI-HOOK-HA strain was generated by transfection of ΔKu80 with linearized pLIC-KI-HOOK-HA-DHFR. KI-HOOK-3Ty strain was generated by transfection of ΔKu80 with linearized pG152-KI-HOOK-3Ty-lox-SAG1_3′UTR-HX-U1-lox. The HOOK-KO strain was generated by transfection of RH with 30 μg of pSAG1::CAS9-GFP-U6::sgHOOK1. At 48 h after transfection green fluorescent protein (GFP)-expressing parasites were sorted into a 96-well plate. HOOK-KO clones were later identified by sequence analysis. The iKD-Myc-hook strain was generated by transfection of RHΔKu80 with 30 μg of pSAG1::CAS9-GFP-U6::sgHOOK2 vector along with purified KOD PCR amplicon using primers against 5′COR-pT8TATi1-HX-tetO7S1myc. Resistant parasites were selected using MPA/Xan. Parasites were cloned by limiting dilution in 96-well plates and plates and then analyzed for the integration and expression of the transgenes by PCR and Western blotting, respectively. KI-FTS-HA and KI-FTS-cMyc strains were generated by transfection of RHΔKu80 parasites with linearized pLIC-KI-FTS-HA-DHFR and pLIC-KI-FTS-cMyc-HXGPRT. APR1-2Ty-DHFR KI-FTS-cMyc and APR1-2Ty-HXGPRT KI-HOOK-HA were generated via transfection of KI-FTS-cMyc and KI-HOOK-HA, respectively, using 30 μg of pSAG1::CAS9-GFP-U6::sgAPR1 vector, along with purified PCR amplicon using specified primers (Table 3) on pLinker-2xTy as the template. KI-FTS-2Ty and KI-HIP-2Ty were generated using 30 μg of pSAG1::CAS9-GFP-U6::sg vector along with purified KOD PCR amplicon using specified primers (Table 3) on pLinker-2xTy as the template. FTS-mAID-3HA and HIP-mAID-3HA strains were generated via transfection of RHΔKu80-Tir1 (referred to as Tir1) with 30 μg of pSAG1::CAS9-GFP-U6::sg vector along with purified KOD PCR amplicon using specified primers (Table 3) on pYFP-mAID-3HA.

Resistant parasites were selected using pyrimethamine (1 μg/mL) or mycophenolic acid (25 μg/mL) and xanthine (50 μg/mL). Parasites were cloned by limiting dilution in 96-well plates and analyzed for the integration and expression of the transgenes by PCR and Western blotting, respectively.

### List of antibodies.

The primary antibodies used in this study were as follows: monoclonal mouse α-Ty (hybridoma BB2, 1:10 IFA, 1:10 WB), mouse α-HA 16B12 (Thermo Fisher; 1:1,000 WB), rabbit α-HA (Cell Signaling Technology; 1:400 IFA, 1:500 WB), mouse α-cMyc (Thermo Scientific; 1:100 IFA, 1:500 WB), rat α-cMyc (Abcam; 1:200 IFA), mouse α-Rab11A (S. Marion lab; 1:250 IFA), α-actin (1:10 IFA, 1:10 WB), α-GAP45 (1:10,000 IFA), α-catalase (1:2,000 WB), α-SAG1 (a generous gift from J.-F. Dubremetz, 1:5 IFA), α-GRA1 (1:3,000 WB; Anawa) α-GRA3 (a generous gift from J.-F. Dubremetz, 1:100 IFA), and α-MIC2 (a generous gift from V. Carruthers; 1:10 IFA, 1:10 WB, α-ARO; 1:1,000 IFA). The secondary antibodies used in this study were anti-mouse and anti-rabbit HRP antibodies (Sigma), Alexa Fluor 680-conjugated goat anti-rabbit antibodies and anti-mouse IgG antibodies, Alexa Fluor 488-conjugated goat anti-rabbit and anti-mouse IgG antibodies, and Alexa Fluor 594-conjugated goat anti-rabbit and anti-mouse IgG antibodies (1:20,000 IFA, 1:20,000 WB; Thermo Fisher).

### Western blot analysis.

Parasites were lysed in radioimmunoprecipitation assay (RIPA) buffer (150 mM NaCl, 1% Triton X-100, 0.5% deoxycholate, 0.1% sodium dodecyl sulfate [SDS], 50 mM Tris [pH 7.5]) using standard procedures and suspended in SDS-PAGE loading buffer (50 mM Tris-HCl [pH 6.8], 10% glycerol, 2 mM EDTA, 2% SDS, 0.05% bromophenol blue, 100 mM dithiothreitol) under reducing conditions. The suspension was subjected to 5 min boiling at 95°C and two sonication cycles. SDS-PAGE was performed according to standard methods. Separated proteins were transferred to nitrocellulose membranes and probed with appropriate antibodies in 5% nonfat milk powder in 0.05% Tween 20-phosphate-buffered saline (PBS). Bound secondary peroxidase conjugated antibodies were visualized using either the ECL system (GE Healthcare) or SuperSignal (Pierce).

### Immunoprecipitation.

A minimum of 5 × 10^8^ parasites were lysed on ice for 30 min in modified RIPA buffer (50 mM Tris-HCl [pH 8.0], 2 mM EDTA, 75 mM NaCl, 0.65% NP-40, 0.005% SDS, protease inhibitors) and centrifuged at 14,000 rpm for 10 min to eliminate cell debris. Then, 20 μL of lysate was kept for Western blotting (input). Anti-cMyc-coated agarose beads (Pierce) or anti-HA coated agarose beads (Pierce) were added to the supernatant overnight. Next, 20 μL of supernatant was kept for WB analysis (flowthrough). After five washes in lysis buffer, bound proteins were eluted by boiling the samples in SDS sample buffer. Samples were subsequently subjected to SDS-PAGE and immunoblotting or mass spectrometry analysis.

### IP-MS/MS.

Parasites (1 × 10^8^ to 5 × 10^8^) were washed once in PBS, lysed in 1 mL of Triton X-100 1%–1× PBS (co-IP buffer) completed with protease inhibitors (protease inhibitor cocktail; Sigma). The lysate was frozen/thawed four times, sonicated, and centrifuged at max speed (21,230 × g) for 10 min at 4°C. The supernatant was incubated with anti-HA (mouse anti-HA [16B12], ENZ-ABS118-1000 [ENZO]) or anti-Ty antibodies (mouse anti-Ty ascites produced in the lab) for 2h at 4°C. Protein A-Sepharose CL-4B beads (GE Healthcare Biosciences) equilibrated in co-IP buffer (20 μL) were then added, and the incubation continued for 2 h at 4°C. Immune complexes were washed 4 times 10 min in 1 mL of co-IP buffer. Beads were resuspended into 50 μL of SDS loading buffer 1×, vortexed, and incubated for 10 min at 95°C, and the eluate analyzed by Western blotting and MS.

### GST pulldown.

The full-length Rab11A was glutathione *S*-transferase (GST) tagged by cloning into a pGEX6p3 vector (Pharmacia). The plasmids expressing GST-Rab11B, GST-Rab5A, and GST-Rab7 have previously described (54). Expression of GST-Rabs in BL21 competent cells was achieved by induction with 1 mM IPTG (isopropyl-β-d-thiogalactopyranoside) at 37°C for 4 h. Bacterial lysates expressing all GST recombinants and GST alone (control) were bound to 100 μL of Protino glutathione agarose 4B beads (Machery Nagel) in GST-lysis/binding buffer (50 mM Tris-HCl [pH 7.6], 1 mM EDTA, 1 mM EGTA, 10 mM β-mercaptoethanol, 150 mM NaCl, 0.5% Triton X-100, and 0.5 mM phenylmethylsulfonyl fluoride [PMSF]) overnight at 4°C. The beads were washed five times with washing buffer A (50 mM Tris-HCl [pH 7.6], 10 mM β-mercaptoethanol, 500 mM NaCl, 0.5% Triton X-100, and 0.5 mM PMSF) and three times with washing buffer B (20 mM Tris-HCl [pH 7.6], 150 mM NaCl, 0.65% NP-40, 0.005% SDS, and 0.5 mM PMSF) sequentially. Beads containing 150 μg of the recombinant proteins and the control GST protein were incubated with a lysate from 0.4 billion wild-type RHΔKu80 or HOOK-HA intracellular parasites, overnight at 4°C. Parasites were lysed using modified RIPA buffer (50 mM Tris-HCl [pH 8.0], 2 mM EDTA, 75 mM NaCl, 0.65% NP-40, 0.005% SDS, and 0.5 mM PMSF). After three washes with the lysis buffer, the proteins bound to the beads were eluted with SDS sample buffer by boiling. The samples were subject to immunoblotting or MS analysis.

### Mass spectrometry and proteomic analysis.

After denaturation at 100°C in 5% SDS, 5% β-mercaptoethanol, 1 mM EDTA, 10% glycerol, and 10 mM Tris buffer (pH 8) for 3 min, protein samples were fractionated on a 10% acrylamide SDS-PAGE gel. The electrophoretic migration was halted as soon as the protein sample entered 1 cm into the separating gel. The gel was quickly stained with Coomassie blue, and five bands, containing the entire sample, were cut. In-gel digestion of gel cuts was performed. An UltiMate 3000 RSLCnano system (Thermo Fisher Scientific) was utilized for separation of the protein digests. Peptides were consequently fractionated onto a commercial C_18_ reversed-phase column (75 μm × 150 mm, 2 μm particle; PepMap100 RSLC column [Thermo Fisher Scientific]; temperature, 35°C). Trapping was performed for 4 min at 5 μL/min, with solvent A (98% H_2_O, 2% acetone nitrile. and 0.1% formic acid). Elution was performed using two solvents A (0.1% formic acid (FA) in water) and B (0.1% FA in acetonitrile) at a flow rate of 300 nl/min. Gradient separation was 3 min at 5% B, 37 min from 5% B to 30% B, 5 min to 80% B, and maintained for 5 min. The column was equilibrated for 10 min with 5% buffer B prior to the following sample analysis. The eluted peptides from the C_18_ column were analyzed by Q-Exactive instruments (Thermo Fisher Scientific). The electrospray voltage was 1.9 kV, and the capillary temperature was 275°C. Full MS scans were acquired in the Orbitrap mass analyzer over *m/z* 300 to 1,200 range with resolution 35,000 (*m/z* 200). The target value was 5.00E + 05. 10 most intense peaks with charge state between 2 and 4 were fragmented in the HCD collision cell with normalized collision energy of 27%, and the tandem mass spectrum was acquired in the Orbitrap mass analyzer with resolution 17,500 at *m/z* 200. The target value was 1.00E+05. The ion selection threshold was 5.0E+04 counts, and the maximum allowed ion accumulation times were 250 ms for full MS scans and 100 ms for tandem mass spectrum. Dynamic exclusion was set to 30 s.

### Proteomic data analysis.

Raw data collected during nanoLC-MS/MS analyses were processed and converted into an “*.mgf” peak list format with Proteome Discoverer 1.4 (Thermo Fisher Scientific). MS/MS data were interpreted using search engine Mascot (version 2.4.0; Matrix Science, London, UK) installed on a local server. Searches were performed with a tolerance on mass measurement of 0.2 Da for precursor and 0.2 Da for fragmented ions, against a composite target decoy database (50,620 total entries) built with three strains of Toxoplasma gondii ToxoDB.org database (strains ME49, GT1, and VEG; release 12.0, September 2014; 25,264 entries) fused with the sequences of recombinant trypsin and a list of classical contaminants (46 entries). Cysteine carbamidomethylation, methionine oxidation, protein N-terminal acetylation and cysteine propionamidation were searched as variable modifications. Up to one trypsin missed cleavage was allowed.

### Microneme secretion assay.

Freshly egressed parasites (±IAA 24 h or ±ATc 70 h) were resuspended in equal volume of intracellular buffer (5 mM NaCl, 142 mM KCl, 1 mM MgCl_2_, 2 mM EGTA, 5.6 mM glucose, 25 mM HEPES [pH 7.2] adjusted with KOH) prior to pelleting at 1,000 × *g* for 10 min. Pellets were subsequently washed in 500 μL of intracellular buffer and repelleted. Pellets were resuspended in 100 μL of serum-free media and incubated with 2% EtOH for 30 min at 37°C. Parasites were pelleted at 1,000 × *g* for 5 min at 4°C. The supernatant was subsequently transferred to new tubes and repelleted at 2,000 × *g* for 5 min at 4°C. Final supernatant (ESAs [excreted secreted antigens]) and pellet fractions were resuspended in SDS sample buffer and subjected to 5 min boiling at 95°C and two sonication cycles prior to immunoblotting. The data are presented as means ± the standard errors of the mean (SEM) of three independent experiments.

### Plaque assay.

Human foreskin fibroblast (HFF) monolayers were infected with 100 μL of serially diluted parasites (1:100, 1:1,000, and 1:10,000) and allowed to proliferate for 7 days with or without IAA. Plaques were fixed in 4% paraformaldehyde–0.05% glutaraldehyde (PFA-Glu) for 10 min, quenched in 0.1 M glycine-PBS, and subsequently stained with crystal violet (Sigma-Aldrich) for 10 min. The data are presented as means ± the SEM of three independent experiments.

### Immunofluorescence assay.

Freshly egressed parasites were inoculated onto HFF coated coverslips. Postinfection (typically 24 to 30 h), the parasites were fixed with 4% PFA or PFA-Glu for 5 to 20 min and quenched in 0.1 M glycine-PBS. Subsequently, the cells were permeabilized 20 min in 0.2% Triton-PBS and blocked for 20 min in 2% bovine serum albumin (BSA)-PBS. Primary antibodies were incubated for 1 h and washed three times in PBS, followed by a 1 h of incubation with secondary antibodies and washed as described previously. Coverslips were mounted onto slides with DAPI-Fluoromount G (Southern Biotech). Confocal images were acquired with a Zeiss confocal laser scanning microscope (LSM800) using a Plan-Apochromat 63× objective with NA 1.4 at the Bioimaging core facility of the Faculty of Medicine, University of Geneva or at the BiCel imaging platform (UMS 2014-US 41-PLBS, Lille). Image analysis and processing were done with ImageJ (Fiji).

### Intracellular growth assay.

Freshly egressed parasites were inoculated onto HFF-coated coverslips. At 24 or 30 h postinfection, the parasites (±IAA) were fixed with PFA-Glu for 20 min and quenched in 0.1 M glycine-PBS. Growth was assessed via immunofluorescence assay staining for both GAP45 (1/:10,000) and GRA3 (1:2,000). Then, 100 vacuoles were counted for three independent experiments. The data are presented as means ± the SEM of three independent experiments.

### Invasion assay.

Freshly egressed parasites (pretreated ±IAA for 24 h) were inoculated onto HFF-coated coverslips. Immediately, the parasites were centrifuged for 1 min at 1,300 rpm, allowed to invade for 30 min at 37°C. After invasion, the coverslips were washed twice in 1× PBS, fixed with PFA-Glu for 20 min, and quenched in 0.1 M glycine-PBS. Invasion was assessed via immunofluorescence assay staining for both SAG1 (1:10) and GAP45 (1:10,000). SAG1 antibody was incubated prior to permeabilization, followed by 5 min of fixation and quenching, and then GAP45 after permeabilization. Finally, 100 parasites were counted for three independent experiments. The data are presented as means ± the SEM of three independent experiments.

### Egress assay.

Freshly egressed parasites were inoculated onto HFF-coated coverslips. At 30 h postinfection (±IAA), the cells were washed in serum-free media, followed by incubation for 12 min in serum-free medium containing BIPPO, A23187, or dimethyl sulfoxide (DMSO). The coverslips were then fixed with PFA-Glu for 20 min and quenched in 0.1 M glycine-PBS. Egress was assessed via immunofluorescence assay staining for both GAP45 (1/:10,000) and GRA3 (1:2,000). A total of 100 vacuoles were counted for three independent experiments. The data are presented as means ± the SEM of three independent experiments.

### Attachment assay.

Freshly egressed extracellular parasites (pretreated ±ATc for 48 h or ±IAA for 24 h, depending on the strain) were counted and resuspended in Endo buffer (44.7 mM K_2_SO_4_, 10 mM Mg_2_SO_4_, 100 mM sucrose, 5 mM glucose, 20 mM Tris, 0.35% BSA [pH 8.2]) containing 1 μM cytochalasin D. A total of 1 × 10^6^ parasites were then seeded onto confluent HFF cells grown on glass coverslips, spun down for 2 min at 1,000 rpm, and incubated for 15 min at 37°C in the presence of 1 μM cytochalasin D. The coverslips were washed with PBS before fixation with PFA 4% for 10 min and then subjected to a red-green assay. Briefly, adherent external parasites were labeled without permeabilization with mouse anti-SAG1 antibodies, followed by secondary anti-mouse antibodies coupled to Alexa Fluor 488. After cell permeabilization with 0.1% Triton X-100, invaded intracellular parasites were detected using rabbit anti-GAP45 antibodies, followed by a secondary anti-rabbit antibody coupled to Alexa Fluor 594. All parasites labeled green-red were considered extracellular, while parasites exclusively red (positive for GAP45) were considered intracellular. At least 100 parasites were counted for each condition performed in technical triplicates. Data represent means ± the SEM from three independent biological experiments.

### Motility assay.

Glass slides were precoated with 100 μg/mL BSA-PBS. Freshly egressed extracellular parasites (pretreated ±ATc for 48 h or ±IAA for 24 h, depending on the strain) were incubated in HHE buffer (HBSS, 10 mM HEPES, 1 mM EGTA) containing either 1 μM ATc or IAA. A total of 1 × 10^6^ parasites were seeded per well, followed by incubation for 15 min at 37°C. Parasites were then fixed with 4% PFA in PBS for 10 min before being incubated with anti-SAG1 antibodies, followed by goat anti-mouse immunoglobulin G secondary antibodies conjugated to Alexa Fluor 488. At least 100 parasites per coverslip were counted for the presence or absence of a SAG1-positive trail. The data are presented as means ± the SEM of three independent experiments.

### Mouse infection experiments.

A group of eight female mice BALB/c (Janvier Labs), aged 8 weeks, were injected intraperitoneally with 250 parasites of the RHΔKu80 or the HOOK-KO strain. A similar experiment was performed using RHΔΔKu80 Tati and Myc-hook iKD ± ATc. In the latter case, the daily drinking water of the mice was supplemented with 0.2 mg/mL of ATc and 5% sucrose to induce depletion of the TgHOOK protein. The survival of the mice was monitored daily for 2 weeks. The mice were euthanized when the endpoints validated by the ethical committee were reached.

### Serial section transmission electron microscopy.

HFF cells infected with parasites were grown in monolayer on round (13 mm in diameter) glass coverslips and were fixed with 2.5% glutaraldehyde (Electron Microscopy Sciences) and 2% PAF (paraformaldehyde) (Electron Microscopy Sciences) in 0.1 M phosphate buffer at pH 7.4 for 1 h at room temperature. Cells were extensively washed with 0.1 M sodium cacodylate buffer (pH 7.4) and postfixed with 1% osmium tetroxide (Electron Microscopy Sciences) and 1.5% potassium ferrocyanide in 0.1 M sodium cacodylate buffer (pH 7.4) for 40 min, followed by 1% osmium tetroxide (Electron Microscopy Sciences) in 0.1 M sodium cacodylate buffer (pH 7.4) alone for 40 min. Cells were then washed in double distilled water twice for 5 min each wash and *en block* stained with aqueous 1% uranyl acetate (Electron Microscopy Sciences) for 1 h. After a 5-min wash in double distilled water cells were dehydrated in graded ethanol series (2 × 50%, 70%, 90%, 95%, and 2 × absolute ethanol) for 3 min each wash. After dehydration, cells were infiltrated with graded series of Durcupan resin (Electron Microscopy Sciences) diluted with ethanol at 1:2, 1:1, and 2:1 for 30 min each and twice with pure Durcupan for 30 min each. Cells were infiltrated with fresh Durcupan resin for an additional 2 h. Coverslip with grown cells faced down was placed on a 1-mm-high silicone ring (used as a spacer) filled with fresh resin which was placed on a glass slide coated with a mold-separating agent. This flat sandwich was then polymerized at 65°C overnight in the oven. The glass coverslip was removed from the cured resin disk by immersion alternately into hot (60°C) water and liquid nitrogen until glass parted.

Laser microdissection microscope (Leica Microsystems) was used to outline the position of parasitophorous vacuoles on the exposed surface of the resin. The selected area was cut out from the disk using a single-edged razor blade and glued with superglue to a blank resin block. The cutting face was trimmed using a Leica Ultracut UCT microtome (Leica Microsystems) and a glass knife. Then, 70-nm ultrathin serial sections were cut with a diamond knife (DiATOME) and collected onto 2-mm single-slot copper grids (Electron Microscopy Sciences) coated with Formvar plastic support film.

Sections were examined using a Tecnai 20 TEM (FEI) operating at an acceleration voltage of 80 kV and equipped with a side-mounted MegaView III CCD camera (Olympus Soft-Imaging Systems) controlled by iTEM acquisition software (Olympus Soft-Imaging Systems).
